# Spatio-Temporal Diffusion Pattern and Hotspot Detection of Dengue in Chachoengsao Province, Thailand

**DOI:** 10.3390/ijerph8010051

**Published:** 2010-12-29

**Authors:** Phaisarn Jeefoo, Nitin Kumar Tripathi, Marc Souris

**Affiliations:** 1 Remote Sensing and GIS Field of Study, School of Engineering and Technology, Asian Institute of Technology, P.O. Box 4, Klong Luang, Pathumthani 12120, Thailand; E-Mails: nitinkt@ait.ac.th (N.K.T.); Marc.Souris@ird.fr (M.S.); 2 Center of Excellence for Vectors and Vector-Borne Diseases, Faculty of Science, Mahidol University, Salaya, Nakhompathom 73170, Thailand; 3 Institut de Recherche pour le Développement (IRD), UMR 190, Marseille, France

**Keywords:** dengue, spatial statistics, temporal analysis, space-time cluster, hotspot, Geographic Information Systems (GIS)

## Abstract

In recent years, dengue has become a major international public health concern. In Thailand it is also an important concern as several dengue outbreaks were reported in last decade. This paper presents a GIS approach to analyze the spatial and temporal dynamics of dengue epidemics. The major objective of this study was to examine spatial diffusion patterns and hotspot identification for reported dengue cases. Geospatial diffusion pattern of the 2007 dengue outbreak was investigated. Map of daily cases was generated for the 153 days of the outbreak. Epidemiological data from Chachoengsao province, Thailand (reported dengue cases for the years 1999–2007) was used for this study. To analyze the dynamic space-time pattern of dengue outbreaks, all cases were positioned in space at a village level. After a general statistical analysis (by gender and age group), data was subsequently analyzed for temporal patterns and correlation with climatic data (especially rainfall), spatial patterns and cluster analysis, and spatio-temporal patterns of hotspots during epidemics. The results revealed spatial diffusion patterns during the years 1999–2007 representing spatially clustered patterns with significant differences by village. Villages on the urban fringe reported higher incidences. The space and time of the cases showed outbreak movement and spread patterns that could be related to entomologic and epidemiologic factors. The hotspots showed the spatial trend of dengue diffusion. This study presents useful information related to the dengue outbreak patterns in space and time and may help public health departments to plan strategies to control the spread of disease. The methodology is general for space-time analysis and can be applied for other infectious diseases as well.

## 1. Introduction

Dengue is the most common mosquito-borne viral disease of humans. In recent years, dengue has become a major international public health concern [1]. The global incidence of dengue fever has grown dramatically in the past 50 years. The disease is transmitted to humans mainly by the mosquitos *Aedes aegypti* and *Aedes albopictus.* Clinical symptoms varying from dengue fever (DF) or classic dengue to dengue hemorrhagic fever (DHF), which may progress into a severe form known as dengue shock syndrome (DSS) [2]. Normally, dengue virus circulating in the blood of viraemic humans gets ingested by female mosquitoes during feeding. The virus then infects the mosquito mid-gut and subsequently spreads systemically over a period of 8–12 days. After this extrinsic incubation period, the virus can be transmitted to other humans during subsequent probing or feeding. Dengue can be a severe, flu-like illness that affects infants, young children and adults, but seldom causes death. The clinical features of dengue fever vary according to the age of the patient. Infants and young children may have a non-specific febrile illness with a rash. Older children and adults may have either a mild febrile syndrome or the classical incapacitating disease with abrupt onset and high fever, severe headache, pain behind the eyes, muscle and joint pains, and rash [3]. Till now, the disease has been reported in over 100 countries located in Africa, the Americas, the Eastern Mediterranean, Southeast Asia and the Western Pacific, which threaten the life of more than 2.5 billion people in urban, periurban, and rural areas of the tropics and subtropics. Dengue fever incidence is high in countries with tropical and warm climates [1]. Causes of increasing dengue transmission may include rapid expansion of urbanization, inadequate water supplies, increased movement of mosquito and human populations within and between countries, and spread of insecticide resistance in mosquito vector populations [4]. In 2010, dengue incidence in several Asian countries constituted a leading cause of pediatric hospitalization [5].

In Thailand, an upward trend in the incidence of dengue has been observed, with acute and severe forms of dengue virus infection, since the first dengue outbreak in 1958 [6]. In 2008, according to dengue surveillance data from the Thai Ministry of Public Health (MOPH), the total numbers of reported cases of dengue infections in Thailand were 43,911, with 46 deaths nationwide, including 18,797 DF cases, 24,455 DHF cases, and 659 DSS cases.

Epidemiological studies of dengue are not easy, for many reasons. The first reason is the strong silent transmission (about 80% of cases display no symptoms). Only severe cases were reported; patients with low or mild symptoms were not considered by the health system. In Thailand as seen in other Southeast Asian countries, the disease can also be confused with others, like influenza, if the diagnostic is only based on symptoms, as the two diseases pose almost the same temporal pattern. The second difficulty correlates with the first one, which concerns with unknown immune status of the population towards dengue infections. Dengue virus have four known serotypes, and each serotype induces a lifelong immunity in recovery cases. Immunity in the population is therefore very important to understand the epidemiology of the disease. The third difficulty lies in the fact that dengue is a vector borne disease, and many factors of outbreaks are related to the vector behavior and its relationship with the environment, like climate, breeding site density probability and vector control, urbanization, human population movement, etc. The presence and density of the vector (mainly *Aedes aegypti* in the urban and peri-urban environment) is difficult to estimate. Climatic factors such as rainfall, temperature, humidity all influence dengue transmission. The high level of humidity during the rainy season makes the survival of the mosquito to be longer [7,8]. By implication Thailand’s rainy season from May to September provides optimal temperatures for *Aedes aegypti* mosquitoes to thrive [9]. Consequently, these conditions facilitate dengue epidemic outbreaks. Moreover, the Thai Meteorological Department (TMD) has reported higher dengue outbreaks in El Niño years. El Niño events in Thailand are actually related to high temperature and low rainfall [10]. Hence a major objective of this study was to identify dengue diffusion patterns with respect to space and time.

GIS can be used to assess and identify potential risk factors involved in disease transmission such as socio-economic, climatic, demographic, and physical-environment variables. GIS technologies have been applied in epidemiological public health studies for many years [11–13]. GIS and spatial analysis are powerful tools in addressing epidemiological problems, allowing the identification of critical areas and variables intimately related to the modulation of the disease dynamics [14,15].

Spatial analyses and statistics, such as spatial autocorrelation analysis, cluster analysis, temporal analysis, are commonly used to highlight spatial patterns of diseases and to test whether there is a pattern of disease incidence in a particular area [16–19]. Recent advances in spatial statistics in GIS have led to a growing interest in the detection of disease clusters or “hotspots” for public health surveillance, in particular for improving the understanding of the growing incidence of dengue fever [20]. Spatio-temporal patterns can provide clues in understanding the dynamics of disease spread. Detection of spatial, temporal and space-time clustering is useful in identifying higher risk areas and times, where disease surveillance and control need to be targeted [21]. For instance, Rotela *et al.* [13] investigated the spreading dynamic of dengue fever outbreak in Tartagal city by Knox’s test method. Cummings *et al.* [22] examined the spatial-temporal dynamics of dengue occurrence in Thailand by applying empirical mode decomposition method to show the existence of a spatial-temporal traveling wave. Maidana and Yang [23] measured the speed of dengue dissemination following the invasion and colonization by only the movement of adult mosquitoes. Tran and Raffy [24] developed model for spatial and temporal dynamics of dengue. Hence, in this study spatial statistical analyses were used to investigate spatio-temporal diffusion patterns of dengue cases.

## 2. Materials and Methods

### 2.1. Study Area: Chachoengsao Province, Thailand

**Figure 1:**
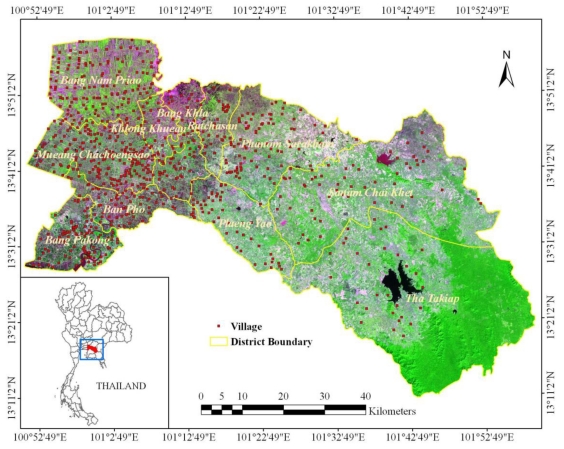
Study area: Chachoengsao province, Thailand.

Among the provinces under the surveillance of MOPH, Chachoengsao, a province in the central part of Thailand near the Bangkok area, had the second highest dengue morbidity rate in Thailand in 2008, with 39.68 cases per 100,000 inhabitants (562 cases), therefore Chachoengsao was selected as the study area (Figure 1) because of the high disease incidence, easy logistics and several types of landscape. Chachoengsao province comprises 11 districts, 93 sub-districts, and 820 villages. The province is located 80 km east of Bangkok, and covers an area of 5,238 square kilometers with geographical location between 13°10′48″N to 13°58′48″N and 100°50′24″E to 101°59′24″E. The province has a population of about 645,000 people (Department of Provincial Administration, 2007). The western part of the province is the low river plain of the Bang Pa Kong River, which is extensively used for paddy cultivation. The eastern part is mountainous, with an average height of more than 100 m above sea level. About 29% of the population are predominantly involved in agricultural activities that take place in an extension of approximately 4,357 square kilometers, including paddy fields, para rubber, sugarcane, and cassava. The average temperature is around 34 °C in the summer season (February–May), 30 °C in the rainy season (May–October), and 28 °C in the cold season (October–February). The average rainfall is approximately 1,283 mm in the summer season, 2,039 mm in the rainy season, and 158 mm in the cold season.

### 2.2. Data

#### 2.2.1. Dengue epidemiological data

Dengue cases data reported in years 1999 to 2007 was used in this study. The data was obtained from the Chachoengsao Provincial Public Health Office (CPPHO), with regard to the number of reported apparent and confirmed dengue cases per village and per day. After the first dengue cases were confirmed, all persons that had visited hospitals in Chochoengsao province with the following symptoms: temperature ≥ 38 °C, headache, arthralgia, and myalgia, were considered as suspects of dengue infection and their cases reported to CPPHO who notify the Bureau of Vector Borne Disease (BVBD), MOPH. Data represented only the patients and were filled in the official form 506 by the CPPHO. The forms provided data for each patient’s age, gender, address, and the dates of the symptoms and for hospital consultation.

#### 2.2.2. Village data

In this study, location data and population data for 820 villages of the province were collected from the Department of Provincial Administration, Thailand. Village point locations were confirmed for accuracy by overlaying on high resolution QuickBird satellite images.

#### 2.2.3. Climatic data

Monthly rainfall (mm), temperature (degree Celsius), and relative humidity (percent) for the years 1999–2007 were obtained from 14 weather stations of Thai Meteorological Department (TMD). Each station provided data for rainfall (mm), minimum/maximum temperature (°C), and relative humidity (%).

### 2.3. Spatio-Temporal Diffusion Pattern

#### 2.3.1. General analysis

For each year, dengue incidence per year by gender (male and female) and age groups was analyzed. Moreover, the gender (male and female) and age groups were also analyzed.

#### 2.3.2. Temporal analysis

Monthly data with dengue cases and with climate data from years 1999 to 2007 was generated. Subsequently, the temporal patterns of dengue cases and incidence were analyzed. Furthermore, the correlation between dengue cases for month t and monthly means of climatic data (rainfall, temperature, and relative humidity) for month t and (t−1) was analyzed. A classic Pearson correlation coefficient was used to assess correlation.

#### 2.3.3. Spatial analysis

Data from all the dengue reported cases was geocoded using village location from the address of the patient. Initial assessment for geographical accuracy at the village level revealed sufficient information to study the spatial pattern of the disease, and allowed us to use the patient address as the location of the infection.

Mapping incidence is the first step in spatial analysis of a disease, but mapping, as always with any ratio, need to be made carefully. Villages with a small number of inhabitants are more variable than villages with high numbers of inhabitants, and ratios may also reflect this difference in statistical variability. While a small population density occurs generally in large areas, mapping reinforces this difference and may give a false view of observed reality. To overcome this problem, an empirical Bayes smoothing (EBS) method based on the idea of pooling information across villages was developed [25]. Essentially, rates were smoothed and thus stabilized by borrowing strength from other spatial units [26]. The dengue incidence rate per year or per month were adjusted by EBS function and converted to the dengue fever morbidity rate (DFMR) [27].

A standard deviational ellipsis (SDE) was used to visualize and compare the major infected areas of the disease. It revealed the directional bias in the point distribution [28]. Furthermore, the SDE parameter for each year was calculated, in order to compare between the years the global position and the spatial extent of the disease.

Global Moran’s I statistic was used to identify characteristics of the global spatial pattern. The global Moran’s I statistic measures the correlation among spatial observations, and allows to find the characteristics of the global pattern (clustered, dispersed, random) among villages [29]. The Moran’s I statistic was used to evaluate autocorrelation in dengue spatial distribution and test how villages were clustered or dispersed in space with respect to their dengue fever morbidity rate. The indices were evaluated by simulation, and considering the original location of the villages [30]. With an infectious disease like dengue, the spatial patterns usually present a strong clustered autocorrelation due to the spatial relationships between cases and the propagation mechanism of the disease, involving distance and neighborhood.

#### 2.3.4. Space-time analysis

Space-time clustering occurs when excess numbers of cases of a disease are observed within small geographical locations over limited periods of time and this cannot be explained in terms of general excesses in those locations or at those times [21]. Understanding, and ultimately being able to predict, the spatio-temporal dynamics of dengue outbreaks at spatial scales ranging from cities to countries and continents is critical to our ability to prevent and control the disease. GIS software and improved analysis techniques provide opportunities to study and model spatio-temporal dynamics of dengue outbreak [22,31,32]. GIS software contains several space-time data analysis tools, which are supposed to be quite useful for the study of spatial epidemiology. This section illustrates the space-time analysis procedures of dengue outbreak. To find how dengue is spreading in space and time, tracking analysis was chosen to create map objects that move or change status with time: temporal data, displaying data, charting temporal data [33]. The data from the 551 dengue cases was considered in the analysis, and further analyzed by the date of onset of symptoms and indicated by space and time dynamics (animation) to see spreading patterns during 153 days from May to September in 2007 of the outbreak (Figure 8).

### 2.4. Hotspot Detection

Hotspot is defined as a condition indicating some form of clustering in a spatial distribution [34]. Hotspot detection can be useful, even if the global pattern is not clustered. Moreover, clusters of cases that occur randomly can also have an influence on the spread of an infectious disease.

Local indicators of spatial association (LISA) can be used to determine locations of clusters or hotspots [35]. The LISA method was carried out in order to find the dengue case hotspot patterns (clustered/dispersed/random) at the local level. The local Getis-Ord Gi^*^ (*d* ), statistics was used to test for statistically significant dengue local autocorrelation, for each year. The local Gi^*^ (*d* ), statistic is useful for determining the spatial dependence of neighboring observations [36–38]. The result expresses the p-value of the calculated Gi^*^ (*d* ), in comparison with the normal distribution of the statistics calculated by simulation [39] (the variability of the local indices is evaluated by simulation and the spatial pattern of the villages is not influencing the result). In this study, adjacency is defined using Thiessen polygon continuity weight file which has been constructed based on villages that share common vertices. 99% significance level (p-value < 0.01) was used to indicate significant clusters of local autocorrelation.

Then, the local Moran’s I value was used to examine the local level of spatial autocorrelation in order to identify villages where values of the DFMR were both extreme and geographically homogeneous [40,41]. This led to identification of so-called dengue hotspots, where the value of the index was extremely pronounced across localities, as well as those of spatial outliers. Thus, the standardized values of DFMR were calculated using the spatial weight matrix that defined a local neighborhood around each geographic unit. The variability of the local indices was evaluated by simulation and taking into account the spatial pattern of the village (clustering of villages have no influence on clustering of incidences). The simulation used permutation of the values among neighbors. The significance level was also set to 99%. Meanwhile, a Moran scatter plot was created with a spatial lag of DFMR on the vertical axis and a standardized DFMR on the horizontal axis (Figure 9). Furthermore, local cluster and hotspot detection were computed on weekly basis by repeating the global analysis for the cases in each week for the epidemic period (May to September, 2007). Mapping location and movement of weekly computed hotspots highlighted the movement pattern of the disease (Figure 10). Next, locations with significant value of clustering were plotted on a map from years 1999–2007 to display the specific locations of dengue hotspots (Figure 11).

### 2.5. Software

Various softwares, namely SavGIS (www.savgis.org), ArcGIS (www.esri.com), GeoDa, SatScan, and SPSS, were used in this study.

## 3. Results

### 3.1. Spatio-Temporal Analysis of Dengue

#### 3.1.1. General analysis

Dengue occurred in most villages of Chachoengsao province, causing severe health problems and financial tolls on the population affected. In 2001, the total number was 1,236 cases, which is the highest recorded incidence for the current decade. After 2002, a gradual decrease was seen in the number of dengue cases until 2007, when another increase occurred. The lowest occurrence was in 1999 (266 cases). As shown in Table 1, in total 5,831 cases were reported, including 3,132 males and 2,699 females. During the highest dengue incidence in year 2001, 626 male and 610 female patients were suspected cases. There were slightly more male patients (53.71%) than female patients. The epidemiological data collected from 1999–2007 was classified into several demographic groups such as gender, area, timing, and age groups.

**Figure 2:**
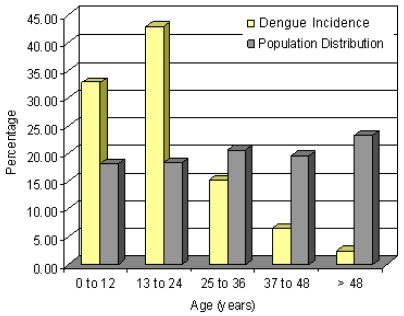
Dengue incidence and percentage population in different age groups (2007).

Disease distribution based on age of the patients was also determined. The age distribution of dengue cases observed was different from the general population age distribution in the province (Figure 2). The highest incidence was in the 13–24 years age group with a percentage of 42.93% (340 cases), while incidence in the 0–12 years age group was 32.82% (260 cases). In the population older than 25 years, the incidence was only 24.25% (192 cases).

#### 3.1.2. Temporal analysis

**Figure 3:**
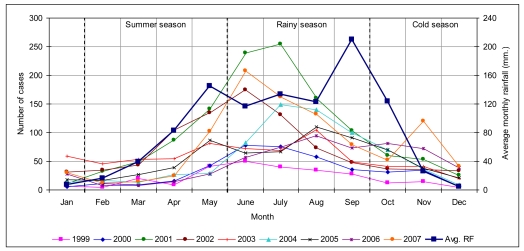
Number of dengue cases and average rainfall on monthly basis in the years 1999–2007.

In 2007, there were 792 suspected dengue patients. The epidemic lasted 20 weeks from 1st of May till 30th of September, coinciding mainly with the rainy season. There were as many as 551 dengue cases spread throughout the province, affecting 0.08% of the total population. Approximately 24.76% of the cases occurred from the 2nd to 4th week of June, with the highest number occurring in Mueang Chachoengsao district (178 cases) and the second highest in Phanom Sarakham district (170 cases). Most cases were in June 2007, with 167 cases, while February 2007 had the lowest number with only nine cases. The comparison of temporal distribution of dengue cases for years 1999 to 2007 is shown in Figure 3.

The dengue temporal distribution in the whole province, with the highest incidence in the rainy season, presented a similar trend every year. The epidemiological trend of dengue follows the three seasons. The disease patterns indicated critical months from May to September that is during the rainy season. The worst incidence was reported in July 2001 with more than 250 cases. Dengue outbreaks generally occurred during the first part of the rainy season, when humidity was higher than average [42,43]. Rainfall (RF), temperature (TEMP), and relative humidity (RH) start to increase in May, consequently the dengue outbreaks reported during the months of June to August, having high rainfalls and humidity. Subsequently number of cases decreases in September when RF and RH were at their highest, but TEMP also showed a decrease.

**Figure 4:**
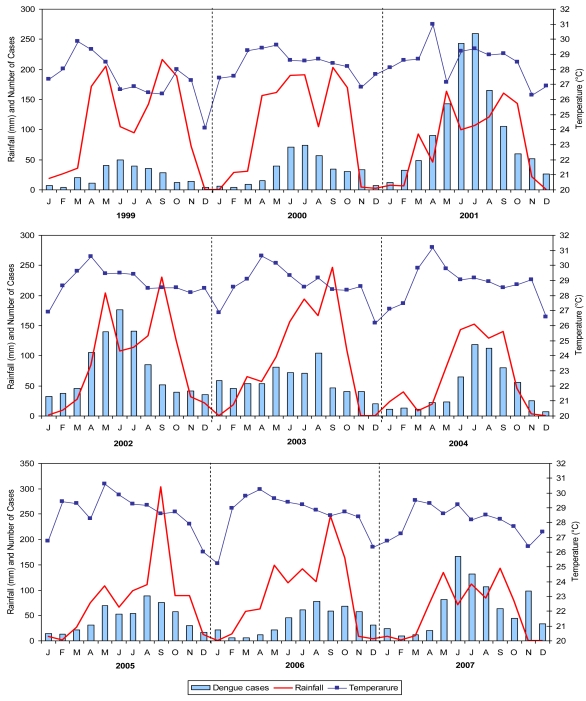
Monthly rainfall (red solid line), temperature (line with symbols) and the total number of dengue cases (histogram) in years 1999–2007.

Furthermore, the number of dengue cases is synchronized and correlated with the rainfall and temperature (with a shift from temperature variation) (Figure 4). It can be seen in Figure 4 that the number of dengue cases were very low level during the cold season and presenting peak during the summer and rainy seasons from May to September, indicating the onset of transmission in summer. Overall, the mean temperature was observed between 25 °C to 31 °C (1999–2007). The average monthly humidity (1999–2007) was 73%.

The Pearson correlation coefficients between cases and climatic data were calculated to analyze relationship between cases number and climatic data, at the same time of occurrence, and with a shift of one month, to let the climatic parameters to have influence on the vector. The highest correlation of RF was seen in 2004 at the same time of occurrence (0.872) and a shift of one month (0.916) (Table 2). The coefficient for 2002 showed high correlation of TEMP with 0.608 and 0.768 at the same time and a shift of one month of occurrence. RH showed highest correlation in 2005 for the same time with 0.840 but in 2006 it presented the highest correlation at a shift of one month with 0.825 in that order. In conclusion, dengue cases were highly correlated with all climatic data, and observed better with one month before shift.

#### 3.1.3. Spatial analysis

Villages are not distributed uniformly in the province, but are strongly clustered (mean of the distances to the nearest village is 1,137 m). Distance for a randomly generated set was 2,957 m (p-value < 0.00001). Mapping disease cases or incidences reflects the spatial distribution of the villages, and need to be carefully analyzed to avoid misinterpretation. However, all the statistical methods used in this study do not depend on the absolute positions of the villages. The results depend only on the relative position of cases compared to non-cases among all villages.

**Figure 5:**
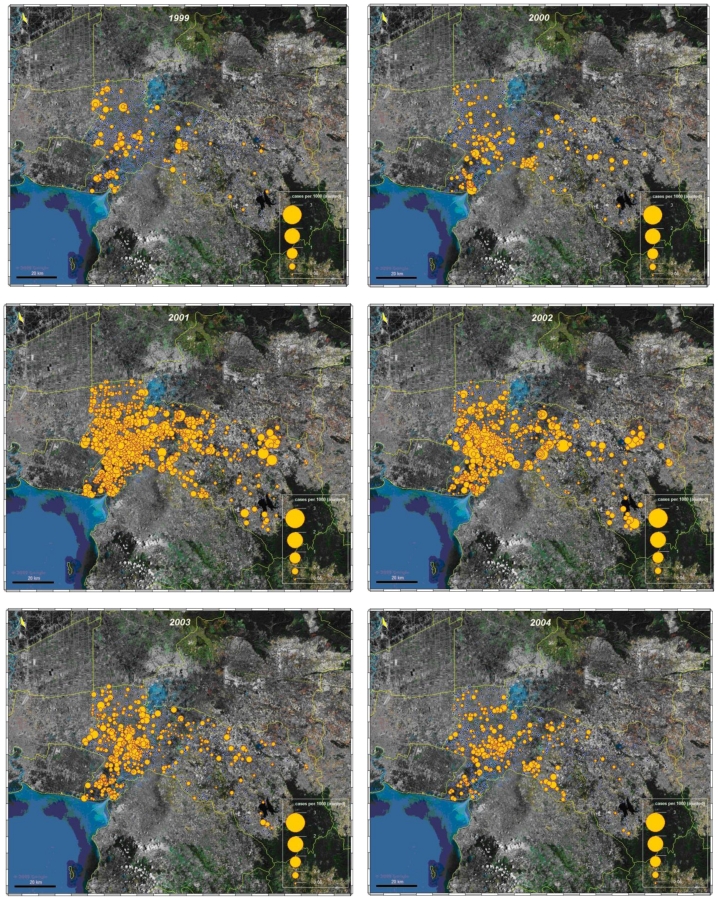

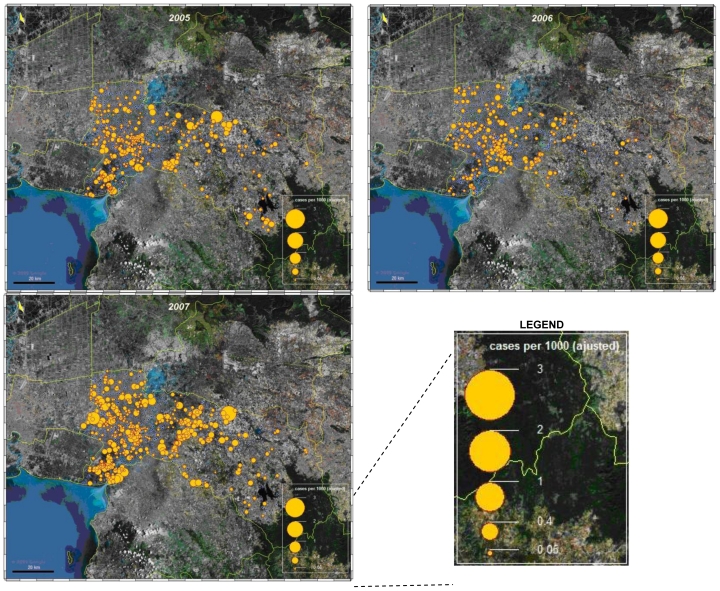
Mapping adjusted incidence of dengue, cases per 1,000 inhabitant and per year.

Mapping adjusted incidence per 1,000 inhabitants and per year allow us to analyze and compare visually the global spatial pattern of the disease. The highest adjusted incidence per 1,000 inhabitants was seen in 2001, while the lowest was in 1999. Maps showed that the disease occurred everywhere in the province, even in villages in the eastern part of the province, which is more rural with a lower density of villages and population (Figure 5).

**Figure 6:**
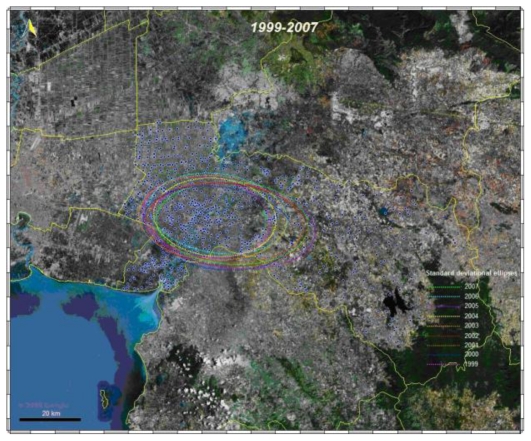
Standard deviational ellipses for each year.

The standard deviational ellipses (SDE) for each year represented the absolute global position and synthesized trend in direction and extent for all infected villages (positive number of cases). SDE of each location was observed predominantly in urban areas (Mueang Chachoengsao, Khlong Khuean, and Bang Khla districts) of the Chachoengsao province. The purpose of the map was to compare the global distribution of infected village for 9 consecutive years. The global pattern was observed almost same in each year, with no significant differences (Figure 6).

The global spatial autocorrelation analysis with Moran index showed that the spatial distribution of DFMR was clustered, for all years (1999–2007) (Table 3). This information is a major finding to suggest public health departments that dengue is occurring in cluster and not spread uniformly or randomly throughout the province. These locations may be considered as hotspots for future strategy to control. The highest of Moran’s I and G-statistic (Z-score) values were confirmed 0.12 and 7.79 respectively in the year 2004. It presented expected clustered pattern for an infectious disease, even at village level.

#### 3.1.4. Space-time cluster analysis

**Figure 7:**
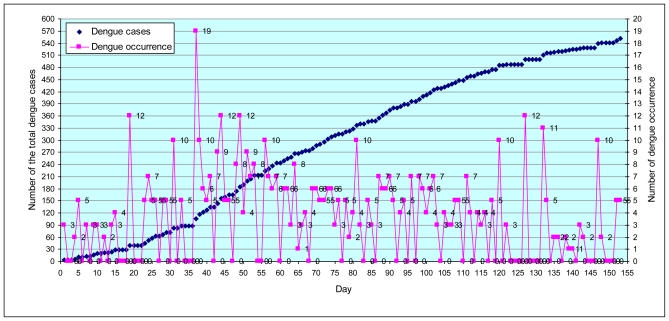
Number of suspected dengue occurrence cases reported (ping color) and number of total dengue cases (dark blue color) during 153 days epidemic (May–September, 2007).

**Figure 8:**
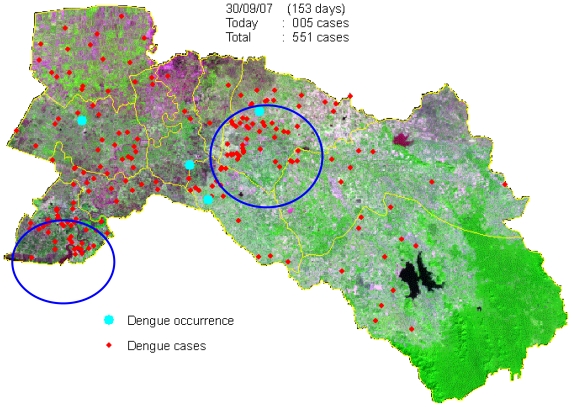
Temporal dynamics in space and time for the 153 days of dengue outbreaks between May to September in the year 2007.

Maps of daily cases were generated, which indicated the dynamics of dengue diffusion through time during 153 days for the months of May, June, July, August, and September for the year 2007. A total of 551 suspected dengue cases were recorded and geo-referenced (Figure 7). It was observed that the temporal dynamics of outbreak continued to the population central district. The highest number of cases per day were 19 dengue cases (day 37: date 06/06/07) in Phanom Sarakham and Bang Nam Priao districts. Spreading patterns analysis was performed by daily (Figure 8).

The highest number of 167 dengue cases was found in the month of June (days 32 to 61) with 30.30% of the suspected dengue cases (Figure 8). It was followed by 130 dengue cases in July (days 62 to 92) or 23.59% of the total number of suspected dengue cases. After this 109 dengue cases occurred in August (days 93 to 123) comprising 19.78% of the suspected dengue cases. Lastly, May and September had 82 cases (days 1 to 31) with 14.88% and 63 cases (days 124 to 153) with 11.43% of the suspected dengue cases in that order. Approximately 54% of the cases occurred in June and July (days 32 to 92). The tracking analysis has shown two cluster patterns (blue color circle), first cluster for south-west (Bang Pakong district) and second cluster for the middle (Phanom Sarakham district) of the study area. This is crucial information that may support the preventive measure by MOPH in controlling the dynamics of outbreak.

### 3.2. Dengue Hotspot Detection

**Figure 9:**
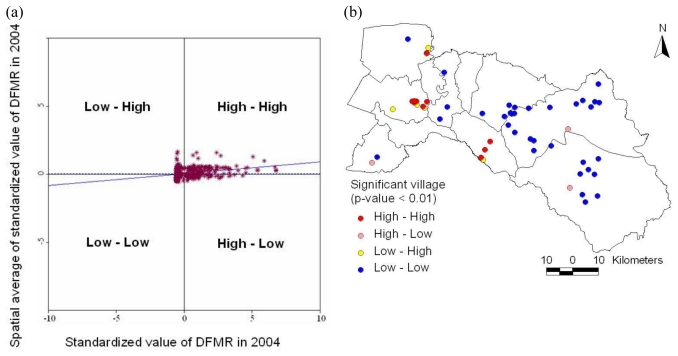
(a) Moran scatter plot matrix and (b) LISA cluster map of DFMR (p < 0.01) for the year 2004.

There were some outstanding spatial clusters of DFMR covering specific locations. The results are presented for the year 2004. The map in Figure 9 shows the locations with significant local indices of spatial association (p-value < 0.01) using local Moran’s I statistics. Those locations were classified by type of association as: the red and blue locations indicating spatial clusters (high surrounded by high, and low surrounded by low), the pink and yellow indicating spatial outliers (high surrounded by low, and low surrounded by high). The standardized values of DFMR in each village were displayed in spatial scatter plot, to contrast observed value with their spatial average (spatially averaged adjacent values), and to detect outliers. The clustered villages with high DFMR (hotspots) were found in the Mueang Chachoengsao, Plaeng Yao, and Bang Nam Priao districts.

**Figure 10:**
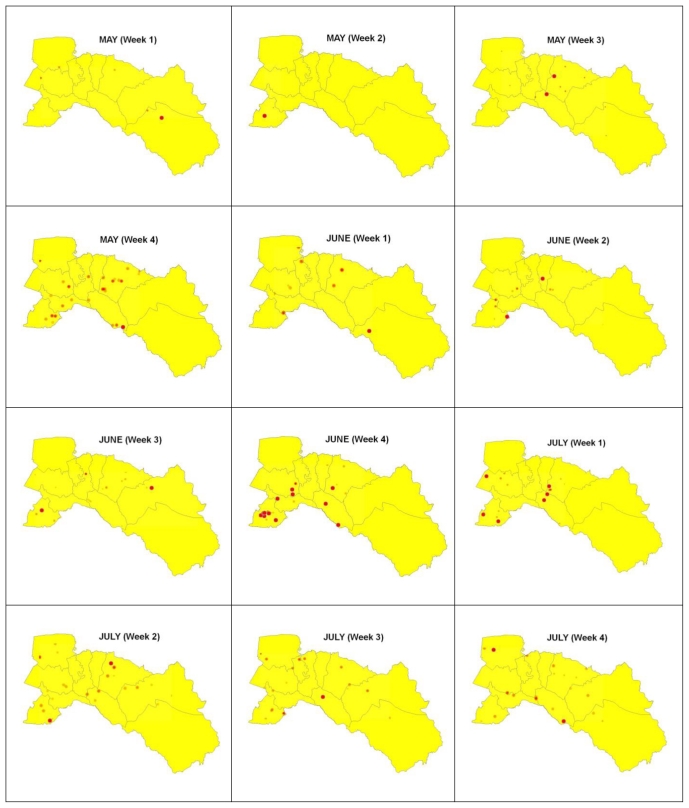

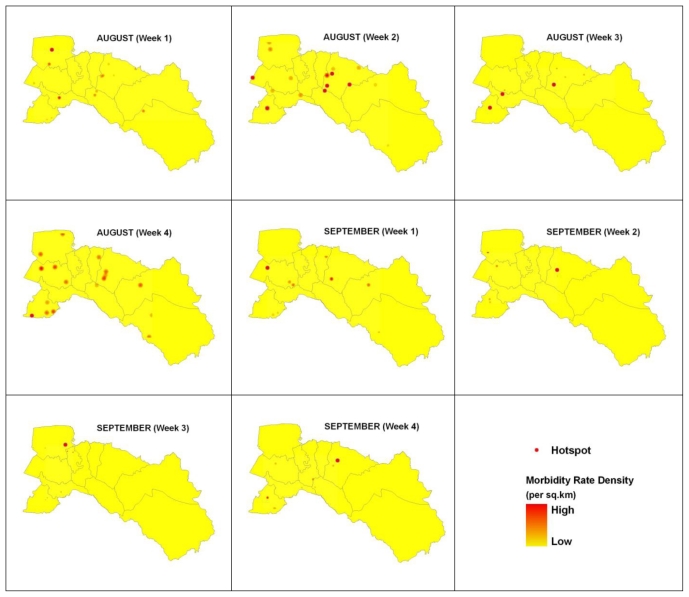
Most likely cluster location in space (red dot) and time for 20 weeks.

**Figure 11:**
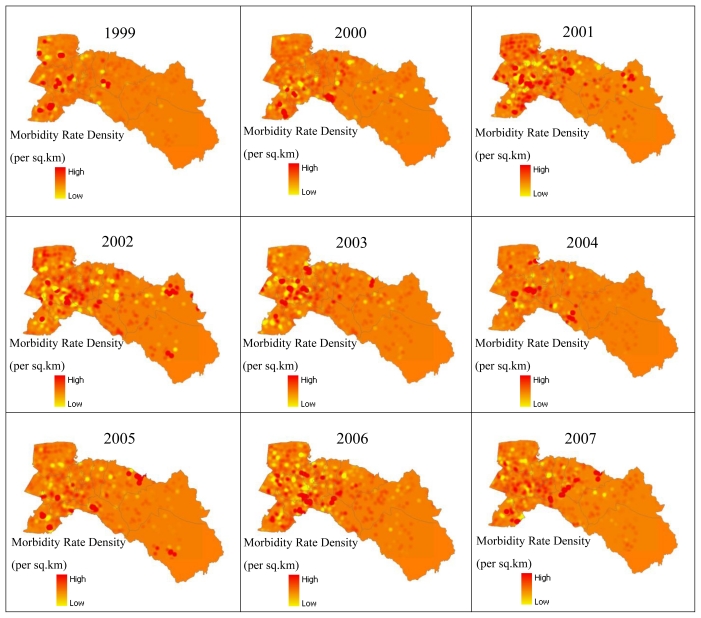
Hotspots of dengue cases for the years 1999–2007.

The dengue hotspots (high-high values) were illustrated by interpolating the values over the space (using Kernel Density Estimation as interpolation method), as shown in Figures 10 and 11. These maps show clear spatial patterns of dengue hotspots. Figure 10 represents the results for 2007 by week. All clusters detected were significant (p-value < 0.01). The dengue epidemic in Chachoengsao province spread rapidly in all the study area during the first weeks and the wide spatial dengue distribution was conserved during the peak of the epidemic, at May week 4 to September week 1. A dengue cases map was built from the cumulative number of cases for each week during the complete epidemic, and confirmed that dengue cases were spread all around the province showing a hotspot with red colored points in the west and middle of the study area. This apparent cluster was due to a notification effect in the native village, where spatial resolution of cases was lower. Once public health services were notified of the outbreak in the province, a faster expansion of the disease was observed compared to the disease expansion in district areas.

The maps of local spatial correlation indices were used to display the hotspots with red zonation (high surrounded by high, respectively) (Figure 11). These maps show clear spatial patterns of dengue that were mostly spread in the west (Bang Nam Priao, Mueang Chachoengsao, Ban Pho, and Bang Pakong districts), and middle (Bang Khla, Khlong Khuean, and Plaeng Yao districts) of the study area from 1999, 2000, and 2003–2007 while in 2001 and 2002 they were determined in the north-east (Sanam Chaikhet district) of the province. The highest density of clustering of hotspots occurred within the urban areas of Mueang Chachoengsao district for the years 1999, 2001–2007, Bang Nam Priao district in 1999, 2001, and 2004, Bang Pakong district were almost spread during 1999–2007 except in 2004, Ban Pho district in 2000, 2001, 2005, and 2006, Plaeng Yao district in 2000, 2002, and 2004–2007, Khlong Khuean district in 2003, 2004, and 2006, Phanom Sarakham district in 1999, 2001, 2003, and 2005–2007, and Sanam Chaikhet district in 2001 and 2002.

## 4. Discussion

Spatial epidemiological research has a long history, but epidemiology studies using GIS has emerged only recently. With the development of computer technology and spatial analysis methods, GIS is becoming more and more important [33]. Monitoring and planning control measures for dengue epidemics have recently become vital to control disease outbreaks. This article aimed at providing useful information on dengue incidences and mapping their patterns and dynamics of diffusion. Spatial autocorrelation analysis proved to be a valuable tool to analyze the spatial patterns change over time.

The study revealed useful information on age group and gender vulnerability to dengue. Incidence of dengue observed to be greater than expected in the 0–24 years old age group and lower in population with less mobility like older than 25 years old. Additionally, several studies confirmed that dengue risk exposure is greater at home because of the endophilic habits of *Aedes aegypti* [13,27,44]. However, clinical symptoms may also be reported to a lesser degree by young people because of better self-recovery ability [13].

Climate also plays important role and it was seen that dengue is generally prevalent in the province of Chachoengsao during the months of May to September. Temporal analysis of climatic factors (rainfall, temperature, and humidity) showed that dengue generally occurs when average temperatures increase, when the humidity is higher than average, and when the rainfall season has already started. As shown, rainfall and relative humidity data of one month before (t−1) showed very high correlation with dengue incidence. Globally, the vector-borne disease and associated vector activity are positively associated with temperature (<40 °C) [45]. There are number of studies in the literature dealing with relationships between temperature and dengue occurrences and dengue vector abundance [10,12,46]. Nakhapakorn and Tripathi [10] reported that the dengue occurrences in Thailand were positively associated with rainfall and negatively associated with temperature and humidity, whereas during the rainy season they were positively associated with rainfall and temperature and negatively with humidity. Similarly, in Taiwan, Wu *et al.* [12] found a positive association between the number of dengue occurrences and the monthly maximum or minimum temperature and the cumulative rainfall with a lag of two months. These observations are coherent with the biology of vectors of viruses. It was shown in many regions that the minimum temperature is the most critical factor for the threshold of mosquito survival and developing rate in sustaining the population density. Likewise, Sriprom *et al.* [46] found dengue virus infection incidence to be positively associated with the monthly minimum temperature, consistent with the literature, and for the extrinsic period as the virus would not amplify in the vector when the temperature becomes less than 18 °C.

Using spatial analysis methods in GIS, the spatial patterns of dengue cases in Chachoengsao province from 1999–2007 were mapped and analyzed. The nature of spatial distribution was found to be clustered in high density population centers. Concerning the empirical Bayes smoothing (EBS) method, raw rates were used to estimate this underlying risk, which reduced differences in population size and in turn addressed variance instability and spurious outliers. In short, rate smoothing presented one way to address this variance instability [47]. The study showed that spatial distribution patterns of dengue cases were significantly clustered, and identified the dengue hotspots in Chachoengsao province. Kernel density estimation illustrated variation in the grouping of dengue areas across the study area, and strongly confirmed the visible pattern on the point location map. Consequently, the village locations were chosen as the best way to analyze the spatio-temporal patterns of the outbreak dynamics over 153 days (May–September) in the year 2007 to study the temporal dynamics in space and time. During the epidemic, there were as many as 551 suspected dengue cases spread throughout the province, affecting 0.08% of the total population. Approximately 24.76% of the cases occurred in June (weeks 2–4). The outbreak dynamics showed a clear non-random pattern of spreading from the first village to other villages each day. The tracking analysis of the disease shows a cluster pattern in the south-west (Bang Pakong district) and in the center (Phanom Sarakham district) of the study area, and also showed how the dengue occurrence locations of disease changed in space and time by movement of days 1 to 153 (Figure 8). Hotspot movement by week did not show clear spread pattern or trend. If related to the temporal distribution of the cases, it showed that concentration of hotspot occurs and then disappears, even when the incidence is high. This result suggested that the disease is spreading locally around foci (radially), with waves of concentration-diffusion process of hotspot. However, the limitation in the study was the dengue cases data. Due to administrative reorganization, some new villages were formed and dengue cases data for these villages was not available for earlier years.

## 5. Conclusions

The results showed that proposed methods and tools can be beneficial for public health officers to visualize and understand the distribution and trends of diffusion patterns of diseases and to prepare warnings and awareness to the masses. Dengue spatio-temporal diffusion patterns and hotspot detection may provide useful information to support public health officers to control and predict dengue spread over critical hotspot areas only rather than for a whole province. This may save time and cost and make public health department efforts more efficient. Public health officers may employ the model to plan a strategy to control dengue by the information received on distribution and hotspots for various months. In future it would be important to have regular daily analysis for several years to converge faster at outbreak locations and be prepared for preventive measures. Some ancillary findings of the study such as influence of climate, which is time dependent, also throw light on its significance. Gender and age groups vulnerability is also an interesting outcome of the study. The methodology is based on notions on general principles of geostatistics and has the potential for application for other epidemics.

## Figures and Tables

**Table 1:** Number of dengue cases classified by gender over the years 1999–2007.

Gender	Year									Percentage (%)
	1999	2000	2001	2002	2003	2004	2005	2006	2007	
Male	143	218	626	475	374	323	306	251	416	53.71
Female	123	162	610	456	316	221	217	218	376	46.29

Total	266	380	1,236	931	690	544	523	469	792	100.00

Source: Chachoengsao Provincial Public Health Office (CPPHO).

**Table 2:** Pearson correlation between climate factor and dengue cases in time series, 1999–2007.

Year	RF		TEMP		RH	

	t	t−1	t	t−1	t	t−1
1999	0.460	0.512	−0.042	0.262	0.631	0.610
2000	0.676	0.839	0.258	0.677	0.647	0.777
2001	0.548	0.516	0.380	0.342	0.539	0.506
2002	0.472	0.346	0.608	0.768	0.158	0.252
2003	0.488	0.296	0.538	0.316	0.544	0.319
2004	0.872	0.916	0.177	0.277	0.682	0.777
2005	0.729	0.481	0.525	0.524	0.840	0.551
2006	0.528	0.836	−0.026	0.325	0.580	0.825
2007	0.446	0.726	0.166	0.487	0.461	0.740

**Table 3:** Global spatial autocorrelation analysis of DFMR.

Year	DFMR		Pattern
	Moran’s I	Z-score	
1999	0.07	5.87	Clustered
2000	0.09	5.45	Clustered
2001	0.06	4.49	Clustered
2002	0.03	3.38	Clustered
2003	0.07	5.87	Clustered
2004	0.12	7.79	Clustered
2005	0.05	2.66	Clustered
2006	0.05	2.79	Clustered
2007	0.07	2.71	Clustered
